# Anastomosis technique for pancreatojejunostomy and early removal of drainage tubes may reduce postoperative pancreatic fistula

**DOI:** 10.1186/s12957-020-02067-4

**Published:** 2020-11-12

**Authors:** Hiromichi Kawaida, Hiroshi Kono, Hidetake Amemiya, Naohiro Hosomura, Mitsuaki Watanabe, Ryo Saito, Yuuki Nakata, Katsutoshi Shoda, Hiroki Shimizu, Shinji Furuya, Hidenori Akaike, Yoshihiko Kawaguchi, Makoto Sudo, Masanori Matusda, Jun Itakura, Hideki Fujii, Daisuke Ichikawa

**Affiliations:** First Department of Surgery, Faculty of Medicine, University of Yamanashi, 1110 Shimokato, Chuo-shi, Yamanashi, 409-3898 Japan

## Abstract

**Background:**

Postoperative pancreatic fistula (POPF) is one of the most serious complications after pancreaticoduodenectomy (PD). Various factors have been reported as POPF risks, but the most serious of these is soft pancreas. To reduce POPF occurrences, many changes to the PD process have been proposed. This study evaluates short-term results of anastomosis technique for PD.

**Methods:**

In total, 123 patients with soft pancreases who had undergone PD at Yamanashi University between January 2012 and August 2020 were retrospectively analyzed. We divided these patients into two groups depending on the time PD was performed: a conventional group (*n* = 67) and a modified group (*n* = 56).

**Results:**

The rate of clinically relevant POPF was significantly lower in the modified group than that in the conventional group (5.4% vs 22.4%, *p* value < 0.001), with there being only one case of POPF in the modified group. There were no cases of POPF-related hemorrhaging in the modified group. On the third day after the operation, the amylase levels in the drainage fluid for the modified group became less than half (1696 vs 650 U/L). Multivariate analysis showed that the modified method was the independent predictors to prevent clinical POPF (*p* value = 0.002).

**Conclusions:**

Our novel anastomosis technique for pancreatojejunostomy reduced POPF in PD, especially in cases where the patient had a soft pancreas.

## Introduction

Pancreaticoduodenectomy (PD) is still the only curative treatment option for malignant and some borderline/benign tumors of the pancreatic head and periampullary region despite the development of various other treatments. With advances in surgical techniques and perioperative management, the operative mortality of PD in high-volume centers has reduced to less than 3% [1–3]. However, incidences of postoperative pancreatic fistula (POPF) have still been reported to be as high as 10% [4–7].

POPF is one of the most serious complications that can result from PD. POPF occurs when there is a pancreatic juice leakage from a surgically exfoliated surface and/or anastomosis. Many previous studies have described several risk factors for POPF, such as gender (male) [8], a high body mass index (BMI) [9], the anastomotic method [6, 10], and the use of an external stent [11]. However, the greatest risks are having a small pancreatic duct (≤ 3 mm) or a soft pancreas [6, 10, 12–17].

In those cases, the anastomosis of the pancreatic duct can be difficult, which may cause anastomotic leakage. Furthermore, overactive exocrine functioning may be deeply involved in the development of POPF, which sometimes causes intraperitoneal abscesses and subsequent lethal hemorrhaging. Therefore, various surgical and perioperative attempts have been made to reduce incidences of POPF. However, there are still many controversies around the anastomosis technique, such as pancreaticojejunostomy (PJ) vs pancreaticogastrostomy [18, 19] and the use of stents [20–22]. Our hospital has conventionally used the PJ method, but our practice has been modified in recent years.

The purpose of this study is to introduce a novel anastomosis technique and analyze its treatment results, including incidences of POPF compared with conventional techniques for patients with a soft pancreas. Furthermore, we analyzed the risk factors of POPF in all cases.

## Materials and methods

### Patients

A total of 237 patients had undergone PD at Yamanashi University between January 2012 and August 2020. Among them, patients who were judged to have a hard pancreas based on intraoperative findings by a surgeon or with a main pancreatic duct diameter of over 2 mm by magnetic resonance cholangiopancreatography (MRCP) were 114. Patients with a past history of pancreatitis were also included in this group. Of these, 13 patients had POPF. In the conventional method, patients with Soft pancreas tended to develop pancreatic fistula. Therefore, to focus on cases of soft pancreas, we excluded them in this study.

The remaining 123 patients who were judged to have a soft pancreas based on intraoperative findings by surgeons, and patients with a main pancreatic duct diameter of 2 mm or less were selected. We divided them into two groups according to the PJ techniques. Fifty-six patients underwent the modified technique (from April 2017 to August 2020; M group), and the remaining 67 patients underwent the conventional technique (from January 2012 to March 2017; C group). The clinical characteristics and pathological examinations were collected from electronic medical records. To supplement the perioperative data, we examined a review of the surgical and anesthetic charts of each patient. This study was approved by the Human Research Ethics Committee of Faculty of Medicine, University of Yamanashi (No. H30232).

### Surgical technique

For the patients with pancreatic cancer, we performed a subtotal stomach-preserving pancreatoduodenectomy. For the patients with other diseases, we performed a pylorus-preserving pancreatoduodenectomy. Portal vein and/or superior mesenteric vein resection was performed in combination with PD in patients with possible or definite tumor invasion. Reconstruction was performed according to the modified Child’s technique. After the jejunal limb was brought up through the antecolic route, we performed an end-to-side PJ first, followed by an end-to-side hepaticojejunostomy and end-to-side gastrojejunostomy. Two closed drainage tubes were routinely placed near the region where the PJ was being performed and at the underside of the hepaticojejunostomy. All the operations were performed by a hepato-pancreato-biliary team.

### Conventional anastomosis for PJ

The outer layer of the end-to-side PJ was sutured in concentric circles centered on the duct-to-mucosa anastomosis. The anastomosis was constructed using 4-0 Vascufil (double-armed polybutester, Tyco Healthcare Co., USA).

**Fig. 1:**
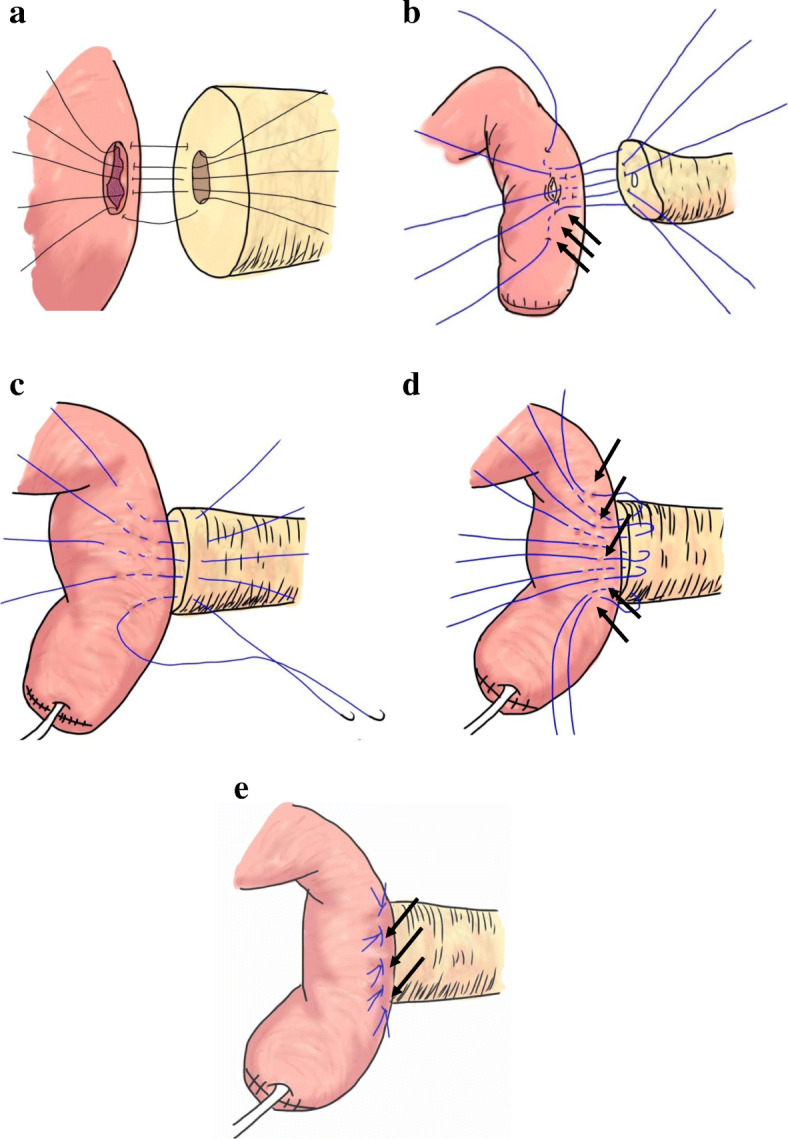
Schemes of PJ. **a** The duct-to-mucosa anastomosis was performed in an end-to-side fashion with eight absorbable interrupted sutures using 5–0 PDS-II with an external stent from the main pancreatic duct. **b** Before the sutures of the duct-to-mucosa were tied, the needle of the 4-0 Vascufil penetrated through the pancreatic parenchyma from the cut surface of the pancreas to the posterior wall. The serous muscle layer of the jejunum was then penetrated in three small steps (so as not to penetrate through all the layers of the wall) from the outside toward the insertion portion of the stent tube. The anastomosis of the posterior wall was performed at three places in total (arrows in **b**). The anastomosis of both the upper and lower edges was performed. The needle of the double-armed 4-0 Vascufil penetrated through the pancreatic parenchyma from the wall of the pancreas to the cut surface near the duct-to-mucosa anastomosis. The serous muscle layer of the jejunum was then penetrated in three steps from near the insertion portion of the stent tube toward the outside (arrows). **c** The anastomosis of the anterior pancreatic wall was performed similarly for both edges. These were performed at three places in total. **d** In the anterior wall and both the upper and lower edges, the needle at the pancreatic side of the double-armed 4-0 Vascufil was sutured at a point 5–8 mm from the lateral side of the previous suture, which penetrated the jejunal seromuscular wall like a triangular mattress suite (arrows). **e** All five sutures were tied gently to prevent tearing of the pancreatic parenchyma. This procedure completely covered the needle holes of the pancreatic wall by the jejunal serosa (arrows)

(1) First, a 5-Fr external stenting tube was inserted into the pancreatic duct through the anastomotic site of the jejunal wall. (2) The duct-to-mucosa anastomosis was performed in an end-to-side fashion with eight absorbable interrupted sutures using 5–0 PDS-II (Johnson and Johnson Co., Tokyo, Japan) and an external stent from the main pancreatic duct (Fig. 1a). (3) Before the sutures of the duct-to-mucosa were tied, the needle of the 4-0 Vascufil was used to penetrate the pancreatic parenchyma from the cut surface of the pancreas to the posterior wall. The serous muscle layer of the jejunum was then penetrated in three small steps so as not to penetrate through all the layers of the wall. This was performed from the outside toward the insertion portion of the stent tube. The anastomosis of the posterior wall was performed at three places in total. (4) The anastomosis of both the upper and lower edges was performed. The needle of the 4-0 Vascufil penetrated through the pancreatic parenchyma from the wall of the pancreas to the cut surface near the duct-to-mucosa anastomosis. The serous muscle layer of the jejunum was then penetrated in three steps (arrows in Fig. 1b) from near the insertion portion of the stent tube toward the outside (Fig. 1b). (5) Then, all three sutures of the posterior wall threads were tied. Subsequently, the sutures of the duct-to-mucosa were tied. (6) Finally, the anastomosis of the anterior pancreatic wall was performed. The needle of the 4-0 Vascufil penetrated through the pancreatic parenchyma from the anterior wall of the pancreas to the cut surface near the duct-to-mucosa anastomosis. The serous muscle layer of the jejunum was then penetrated in three steps from near the insertion portion of the stent tube toward the outside. These procedures were performed at three places in total (Fig. 1c).

### Modified anastomosis: triangular mattress suite method

We made changes to the anastomosis of the anterior pancreatic wall and both the upper and lower edges. The needle at the pancreatic side of the double-armed 4-0 Vascufil was sutured at a point 5–8 mm to the lateral side of the previous suture, which penetrated the jejunal seromuscular wall like a triangular mattress suite (arrows in Fig. 1d). Then, all five sutures were tied gently to prevent the tearing of the pancreatic parenchyma. This procedure completely covered the needle holes of the pancreatic wall with the jejunal serosa (arrows in Fig. 1e).

### Postoperative management

Prophylactic somatostatin analogs were not administered to prevent POPF. The amylase level in the drainage fluid (D-Amy) was routinely measured on postoperative days (PODs) 1 and 3. The drainage tube was removed on POD 3 if the drainage fluid was clear regardless of the amount of drainage fluid or D-Amy, indicating that no bacterial infection existed. POPF was diagnosed according to the International Study Group of Pancreatic Fistula criteria [4].

### Evaluated factors

The following factors that may be associated with the formation of POPF were analyzed in this study: sex, age, BMI, white blood cell (WBC) count in peripheral blood, C-reactive protein (CRP), serum albumin, hemoglobin A1c (HbA1c), operative time, volume of blood loss, intraoperative blood transfusion, D-Amy, and surgical procedures.

### Statistical analysis

Data were expressed as the mean ± standard deviation. Patient characteristics and intraoperative and postoperative factors between the two groups were compared using Chi-square statistics, Fisher’s exact test and the Mann-Whitney *U* test. Multivariate logical regression analyses were conducted to identify independent risk factor for POPF. Significance was defined as a *p* value < 0.05. The statistical analyses were performed using SPSS version 23.0 software (SPSS Inc., Chicago, IL, USA).

## Results

### Patient characteristics

There were no significant differences in sex, median age, serum albumin and HbA1c between the C group and M group. BMI was higher in the M group, but this was not significant. In intraoperative findings, volume of blood loss and frequencies of blood transfusion were not significant. However, for the mean operation time, the M group was significantly longer than the C group. The length of hospital stay was significantly shortened in modified group. Patient characteristics are shown in Table 1.

**Table 1:** Patients’ characteristics and pathologic and operative details

	Conventional (*n* = 67)	Modified (*n* = 56)	*p* value
Male/female	44/23	36/20	0.874
Age (range)	70 (14–86)	71 (31–87)	0.8
BMI (kg/m^2^)	21.8 ± 0.3	23.0 ± 0.5	0.06
Albumin (g/dL)	4.6 ± 0.6	4.0 ± 0.1	0.384
HbA1c (%)	5.8 ± 0.1	5.8 ± 0.1	0.959
Preoperative CRP (mg/dL)	0.4 ± 0.1	0.4 ± 0.1	0.684
Preoperative biliary drainage (yes/no)	27/40	26/30	0.498
Disease			
CBD cancer	24 (35.8%)	20 (35.7%)	
Ampullary tumor	12 (17.9%)	9 (16.1%)	
Duodenal tumor	5 (7.5%)	1 (1.8%)	
Pancreatic cancer	5 (7.5%)	12 (21.4%)	
PNEN	10 (14.9%)	2 (3.6%)	
IPMN	7 (10.4%)	6 (10.7%)	
SPN	2 (3.0%)	3 (5.4%)	
SCN	1 (1.5%)	0	
Metastatic cancer	1 (1.5%)	0	
Other disease	0	3 (5.4%)	
Operative time (min)	450 ± 9	497 ± 14	0.004
Blood loss (ml)	772 ± 64	642 ± 72	0.181
Hospital stays	33.5 ± 1.8	28.4 ± 1.0	0.023

*BMI* Body mass index, *HbA1c* Hemoglobin A1c, *CBD* Common bile duct, *PNEN* Pancreatic neuroendocrine neoplasm, *IPMN* Intraductal papillary mucinous neoplasm, *SPN* Solid pseudopapillary neoplasm, *SCN* Serous cystic neoplasm, *BTF* Blood transfusion

### Comparisons of the postoperative laboratory data and amylase levels in the drainage fluid

There were no significant differences in the WBC count or CRP on PODs 1 and 3 between the C group and M group.

The D-Amy of the M group on POD 1 was similar to the C group (the C group vs the M group, 7738 ± 1544 vs 5122 ± 869 U/L). However, the D-Amy of the M group on POD 3 became less than half of that measured in the C group (1696 ± 914 vs 650 ± 133 U/L) (Table 2).

**Table 2:** Comparisons of the postoperative laboratory data and amylase levels in the drainage fluid

	Conventional (*n* = 67)	Modified (*n* = 56)	*p* value
WBC on POD 1 (/μL)	10548 ± 358	11292 ± 468	0.202
WBC on POD 3 (/μL)	9282 ± 435	9794 ± 416	0.404
CRP on POD 1 (mg/dL)	8.5 ± 0.3	9.1 ± 0.4	0.195
CRP on POD 3 (mg/dL)	13.8 ± 0.9	14.5 ± 0.8	0.572
D-Amy on POD 1 (U/L)	7738 ± 1544	5122 ± 869	0.166
D-Amy on POD 3 (U/L)	1696 ± 914	650 ± 133	0.315

*WBC* White blood cell, *CRP* C-reactive protein, *D-Amy* amylase level in the drainage fluid

### Incidence of POPF

The date of the POPF instances is shown in Table 3. The rate of clinically relevant POPF was significantly lower in the M group than in the C group (5.4% vs 22.4%, *p* value < 0.001), having only one case of POPF in the M group. Furthermore, although there were three cases of POPF-related hemorrhaging in the C group, there were no such cases in the M group. Mortality within 90 days was zero in both groups.

**Table 3:** Comparison of the incidence of postoperative pancreatic fistula

	Conventional (*n* = 67)	Modified (*n* = 56)	*p* value
POPF			
Grade B or C	15 (22.4%)	3 (5.4%)	*<* 0.001
None or biochemical leakage	52	53	

*POPF* Postoperative pancreatic fistula

### Predicting factors and risk factors of POPF

Univariate analysis showed that men, high-BMI patients, and conventional methods were significantly associated with clinically relevant POPF. Multivariate analysis also showed that men and high-BMI patients were independent risk factors for POPF, and the modified method was the independent predictors to prevent clinical POPF (*p* value < 0.001) (Table 4).

**Table 4:** Predicting factors and risk factors of postoperative pancreatic fistula in all cases

	Univariate analysis			Multivariate analysis		
	None or biochemical leakage (*n* = 105)	POPF B and C (*n* = 18)	*p* value	Odds ratio	95% CI	*p* value
Male/female	64/41	16/2	0.059			
Age (range)	69 (14–87)	68 (30–83)	0.775			
BMI (kg/m^2^)	22.0 ± 0.3	24.2 ± 0.5	< 0.001	0.699	0.568–0.862	< 0.001
Albumin (g/dL)	4.0 ± 0.5	4.2 ± 0.4	0.019	0.993	0.285–3.460	0.992
HbA1c (%)	5.8 ± 0.1	5.8 ± 0.1	0.705			
Operative time (min)	470 ± 9	500 ± 18	0.179			
Blood loss (ml)	736 ± 55	615 ± 63	0.364			
Conventional/modified	52/53	15/3	< 0.001	0.083	0.018–0.388	0.002

## Discussion

The rate of clinical POPF is still high at approximately 10–20% after PD [23–27], and the most important risk factors are soft pancreases and non-dilated main pancreatic ducts [28, 29]. Therefore, we focused on patients with soft pancreas and main pancreatic duct diameters of 2 mm or less when revising our techniques for the operation.

In our hospital, the rate of POPF from the conventional method was almost identical to that in previous reports (22.4%). However, the rate of POPF reduced significantly with the modified method, only occurring in 3 of every 56 cases (5.4%). No patient has sepsis, postoperative intervention, or readmission in the M group. Moreover, multivariate analysis showed that the modified method was an independent factor for preventing clinical POPF.

Of the 111 cases excluded in this study, 62 belonged to the C group. Of the 54 cases included in the M group, POPF was observed in 5 cases, and 1 of them was a non-stent case. In all cases including these, M group significantly reduced POPF compared to C group (C group, 17.8%; M group, 7.4%; *p* value = 0.018).

We routinely measured amylase levels in the D-Amy on PODs 1 and 3. Although the median D-Amy of the M group on POD 1 was similar to that of the C group, the median D-Amy of the M group on POD 3 had reduced to one third of the C group levels. This result suggests that our new method prevents the leakage of pancreatic fluid more effectively than the conventional method.

In recent years, a transpancreatic U-suture technique has been devised by Blumgart et al. [30]. Because of the simple method and excellent results, this method has been validated by many surgeons [31, 32], and several surgeons have tried this modified method of novel anastomosis and reported the treatment results [28, 33–36]. This method prevents the tear of pancreatic parenchyma and the jejunum wall, which could be in close contact with the pancreatic cut surface. On the other hand, a transpancreatic U-suture technique might reduce blood flow in the pancreatic stump. Furthermore, if a transpancreatic U-suture is placed from the cranial section of the main pancreatic duct to the caudal section, it may cause stenosis of the main pancreatic duct.

On the other hand, our procedure does not include the problems listed above. That is, the pancreas and jejunum are sutured concentrically around the main pancreatic duct, so there is no concern about decreased blood flow of the pancreas. Main pancreatic duct stenosis can be avoided as the suture does not tighten the main pancreatic duct. By suturing in a concentric shape, the jejunum wall can have close contact with the whole pancreatic cut surface. Furthermore, the needle-penetrated holes in the anterior wall including both the upper and lower edges’ walls of the pancreas can also be widely covered with the jejunum serosa by using triangular mattress suite method. As the needle-penetrated holes in the dorsal pancreatic wall are covered by the splenic vein and soft tissue, it does not cause POPF.

Although stent usage is also controversial, we routinely insert the external stent tube in the main pancreatic duct for patients with soft pancreases to prevent pancreatic trypsin from corroding the anastomotic site during the early period after surgery [20–22]. Also, the drainage tube was removed on POD 3 if the drainage fluid was clear. The timing of the drain removal is not defined. However, a previous study reports improved outcomes with early drain removal after pancreatoduodenectomy [37], and the prolonged placement of a drain might be a major cause of POPF as retrograde intra-abdominal infection may occur [38, 39].

To prevent clinical POPF, pancreato-biliary surgeons have tried various methods and reported their treatment results, including pancreaticoenterostomy [18, 19, 28, 33–36], the use of the pancreatic duct stenting [20–22], the management of the drainage tube [37–39], and somatostatin analogs [40–42]. However, the efficacy of these methods is still controversial. Although the present study has some limitations that were analyzed retrospectively and only presented data from a single institution, our surgical procedure and perioperative management have the possibility to reduce POPF.

## Conclusions

We introduced a novel anastomosis technique for PJ. Although the present study has the limitations of only being based at a single institution, our surgical procedure and the early removal of the drainage tubes may reduce POPF in PD for patients with soft pancreases.

## Data Availability

The datasets generated during and/or analyzed during the current study are available from the corresponding author on reasonable request.

## Abbreviations

PD: Pancreaticoduodenectomy
POPF: Postoperative pancreatic fistula
BMI: Body mass index
PJ: Pancreaticojejunostomy
MRCP: Magnetic resonance cholangiopancreatography
D-Amy: The amylase level in the drainage fluid
POD: Postoperative day
WBC: White blood cell
CRP: C-reactive protein
HbA1c: Hemoglobin A1c
